# A lack of a definite correlation between male sub-fertility and single nucleotide polymorphisms in sperm mitochondrial genes *MT-CO3*, *MT-ATP6* and *MT-ATP8*

**DOI:** 10.1007/s11033-022-07884-2

**Published:** 2022-09-06

**Authors:** Mayyas Saleh Jaweesh, Mohamad Eid Hammadeh, Fatina W. Dahadhah, Mohammad A. Al Smadi, Mazhar Salim Al Zoubi, Manal Issam Abu Alarjah, Houda Amor

**Affiliations:** 1 Department of Obstetrics & Gynaecology, Saarland University, Homburg, Saar Germany; 2 Department of Basic Medical Sciences, Faculty of Medicine, Yarmouk University, Irbid, 21163 Jordan

**Keywords:** Male sub-fertility, CO3, ATP6 and ATP8

## Abstract

**Background:**

An inability of a man to conceive a potentially fertile woman after a year of unprotected intercourse is defined as male infertility. It is reported that 30–40% of males in their reproductive years have abnormalities in sperm production, either qualitatively or quantitatively, or both. However, genetic factors result in up to 15% of male infertility cases. The present study aimed to analyze the possible correlations between sub-fertility and polymorphisms in sperm mitochondrial CO3, ATP6 and ATP8 genes in sub-fertile men.

**Methods and results:**

For 67 sub-fertile and 44 fertile male samples, Sanger sequencing of selected mitochondrial DNA genes was done. A total of twelve SNPs in the MT-CO3 gene: rs2248727, rs7520428, rs3134801, rs9743, rs28358272, rs2853824, rs2856985, rs2854139, rs41347846, rs28380140, rs3902407, and 28,411,821, fourteen SNPs in the MT-ATP6: rs2001031, rs2000975, rs2298011, rs7520428, rs9645429, rs112660509, rs6650105, rs6594033, rs6594034, rs6594035, rs3020563, rs28358887, rs2096044, and rs9283154, and ten SNPs in the MT-ATP8: rs9285835, rs9285836, rs9283154, rs8179289, rs121434446, rs1116906, rs2153588, rs1116905, rs1116907, and rs3020563 were detected in the case and control groups at different nucleotide positions. Only the rs7520428 in the MT-CO3 and MT-ATP6 showed a statistically significant difference between sub-fertile and fertile groups in the genotype’s and allele’s frequency test (P < 0.0001 for both).

**Conclusion:**

The results of our study suggest that male sub-fertility is linked with rs7520428 SNP in MT-CO3 and MT-ATP6. The studied polymorphic variations in the MT-ATP8 gene, on the contrary, did not reveal any significant association with male sub-fertility.

**Supplementary Information:**

The online version contains supplementary material available at 10.1007/s11033-022-07884-2.

## Introduction

Male infertility is related to about 20–70% of infertile couples [1]. It is reported that 30–40% of males in their reproductive years have abnormalities in sperm production, either qualitatively or quantitatively, or both. Oligozoospermia (low sperm count) and asthenozoospermia (low motility) are linked to male infertility in almost half of the cases [2]. Nevertheless, up to 15% of male infertility cases are due to genetic causes [3]. Although some of the related genes are yet unidentified, 40% of idiopathic male infertile cases are attributed to genetic predisposition [4]. Despite the possible role of nuclear genetic variants in male infertility, the sperm mitochondrial DNA genetic alterations are expected to have a significant impact on male infertility or certain subtypes of male infertility such as Asthenozoospermia [5, 6].

The mitochondrial DNA has only exons but no introns, which makes the genetic alteration in the mtDNA more deteriorating which could adversely affect the function of the mitochondrial cellular respiration and eventually a nonfunctional sperm. In Addition, the sperm mtDNA molecule has a very limited and basic repair mechanism because it lacks histones. Collectively, these unique features of the sperm mtDNA make it a vulnerable molecule to any genotoxic agent [7]. Reactive oxygen species (ROS) are by-products of the mitochondrial respiratory chain, the mutation rate generated by ROS is 10–100 times greater than nuclear DNA because the mitochondrial DNA is located in the mitochondrial matrix [8]. The mammalian sperm consists of approximately 80 mitochondria located in the midpiece of the sperm, ensuring proper function of the flagella and normal sperm motility for an efficient fertilization process [9, 10]. Therefore, any genetic alteration of this limited number of mitochondria can be related to the improper function of the sperm [11].

Several studies have reported that mitochondrial dysfunction has a significant impact on sperm structure and motility [12, 13]. In addition, several point mutations and deletions in the mtDNA have been reported in different infertile males [2, 14]. It has been observed that mutations within the mtDNA polymerase gene (POLG) are associated with infertility in men [15]. Moreover, large-scale mtDNA mutations were detected in patient groups with Asthenozoospermia [6, 16].

It was reported that mutations and polymorphisms in the ATP6 and ATP gene sequences are associated with breast cancer especially those of missense type. They cause mitochondrial function disruption [17]. (Grzybowska-Szatkowska, Ślaska, Rzymowska, Brzozowska, & Floriańczyk, 2014). Additionally, Kytövuori and her team in 2016 has reported a novel mutation m.8561C > G in MT-ATP6/8 in an overlapping region and they suggested that this pathogenic mutation is associated with cerebellar ataxia, diabetes mellitus, peripheral neuropathy, and hypogonadotropic hypogonadism and it causes impaired assembly and decreased ATP production of complex V [18]. A novel mutation of the MT-CO3 m.9396 G > A was found to affect the amino acid sequence in the complex IV of the mitochondrial respiratory chain in a patient with mitochondrial encephalomyopathy, lactic acidosis, and stroke-like episodes (MELAS) [19].

Patients with mutations in the MT-ATP6 gene, which codes for a major part of the Fo proton channel, were identified with diseases caused by ATP synthase failure [20]. A common deletion referred to as class I deletion, flanked by 13-bp direct repeats at the following positions 13,447–13,459 and 8470–8482. This deletion removes genes coding for mitochondrial genes ND3, ND4, ND4L and some parts of ND5 as well as ATP6 and CO3 [16].

Consequently, the current study was conducted to examine a possible association between the MT-CO3, MT-ATP6 and MT-ATP8 genes variants and sperm parameters related to male sub-fertility.

## Materials and methods

### Patients recruitment and sample collection

One hundred and eleven individuals were included in this study including fertile and sub-fertile patients visiting the fertility clinic. Participants provided both oral and written permission by signing an informed consent form before sample collection. All volunteers of the case and control group provided an accurate medical history, including age (25–55 years old), smoking habits, chronic medical conditions, prescription medicine use and if there is a history of varicoceles. Semen samples were collected from candidates by masturbation using sterile containers after three days of sexual abstinence.

The control group included 44 men with a normal semen analysis (Concentration: 15 × 10^6^ spermatozoa/ml, total motility: ≥ 40%, progressive motility: ≥ 32%, and normal morphology: ≥ 4%) according to WHO guideline 2010, while the sub-fertile group included 67 men with abnormal semen analysis (Concentration < 15 × 10^6^ spermatozoa/ml, motility < 40%, progressive motility < 32%), [Oligozoospermia, n = 10, Asthenozoospermia, n = 23, Teratozoospermia, n = 19, OAT, n = 15].

Patients with a clinical history of chronic diseases such as blood pressure and diabetes, varicoceles, undergoing chemotherapy or radiotherapy, involved in surgery in the reproductive system, or diagnosed with hypogonadotropic hypogonadism (hormonal disorder) and genetic disorders (Klinefelter's syndrome or Y-chromosome microdeletion) were not included in the study.

The name of the committee that ethically approved this study is Jordanian Royal Medical Services-Human Research Ethics Committee The approval number is F3/1/ Ethics Committee /9126.

### Extraction of mitochondrial DNA

Before DNA extraction all semen samples were washed and purified using a density gradient procedure (45% and 90%) (PureCeption, Cooper surgical, Denmark) as described before [6]. Briefly, Samples were layered over the upper layer of the discontinuous gradient media, then centrifuged at 250 g for 20 min. Next, a sperm washing medium (Global Total HEPES media with HSA) was used to wash the sperm pellet (Cooper surgical, Denmark). A drop of the sperm solution was then examined by microscopic examination to assure the absence of non-sperm cells.

Mitochondrial DNA isolation was carried out in two steps. The QIAamp DNA Mini Kit (QIAGEN, Germany) was used first to isolate the whole genomic DNA along with the mitochondrial DNA (The extraction was done according to the instructions recommended by the kit).

In the second step, we used the REPLI-g Mitochondrial DNA Kit (QIAGEN, Germany) to isolate and amplify only mitochondrial DNA from the samples (The extraction was done according to the instructions recommended by the kit) [5, 6].

### Polymerase chain reaction (PCR)

Primers used for the amplification of the *MT-CO3, MT-ATP6* and *MT-ATP8* genes were designed using the UCSC website and the PRIME 3 software. They were ordered from the Microsynth Seqlab company (Göttingen, Germany) and based on the human mitochondrial sequence; accession number NC_012920, provided by the National Centre of Biotechnology Information (Table 1).

**Table 1:** Forward and Reverse primers for MT-COX3, MT-ATP6, and MT-ATP8 genes and the PCR program used for amplification

Gene	Primer direction	Sequence (5′ ≥ 3′)	Product size
MT-CO3	MT-CO3 forward -F	CCTAGAAATCGCTGTCGCCT	974 bp
	MT-CO3 reverse -R	AAGGCTAGGAGGGTGTTGAT	
MT-ATP6	MT-ATP6 forward -F	CCTCCCTCACCAAAGCCCAT	786 bp
	MT-ATP6 reverse -R	GGTCATGGGCTGGGTTTTACTA	
MT-ATP8	MT-ATP8 forward -F	CCCCTCTAGAGCCCACTGTAA	343 bp
	MT-ATP8 reverse -R	GTGGGGATCAATAGAGGGGGA	

A 30 µl PCR reaction mixture was prepared using MyTaqTMHS Red Mix Kit (Bioline, UK) according to the manufacturer’s instructions. Then, the amplification was performed on the thermocycler (C1000TM Thermal cycler, Bio-Rad, United States) using the program listed in Table 1. To verify the presence of the PCR product, 5 µl of the PCR samples were run on gel electrophoresis using 2% and 1.5% agarose (according to the size of the product) along with DNA Ladder (0.1–10.0 kb) (NE Biolabs, USA). They were run at 75 V for 1 h in 1 × TAE buffer then a red-safe stain was used to stain gels, and finally the DNA was imaged by Molecular Imager® Gel Doc™ XR (BIO-RAD, USA).

### Sanger sequencing

**Fig. 1:**
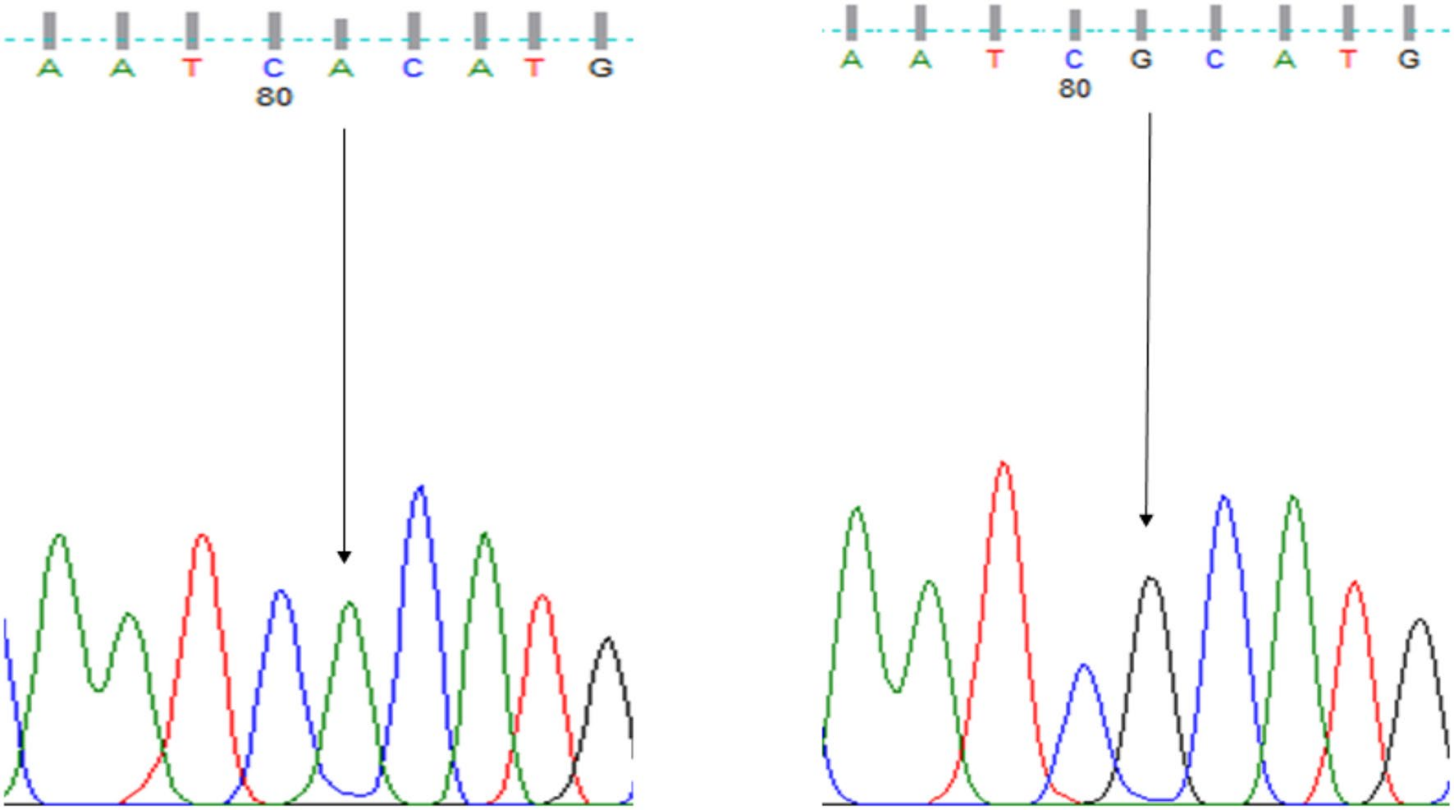
Sequencing electropherogram results (AA, GG) of the rs7520428 of *MT-ATP6* The nucleotide transition at position 634,390 (A > G)

Samples were purified and sequenced using the Sanger sequencing method by Seqlab (Sequencing Laboratories, Göttingen, GmbH). Mutation surveyor software was used to identify the SNPs of the *MT-CO3, MT-ATP6 and MT-ATP8*. Thereafter, all resulting SNPs were genotyped using finch TV (Fig. 1).

### Statistical analysis

Comparing genotypes and allele frequencies between the sub-fertile (case) and fertile (control) groups were conducted by using the Chi-square and Fisher's exact tests The genotype frequencies and statistically significant variations from the equilibrium were determined using the Hardy–Weinberg equilibrium test on the detected SNPs. To compare allele frequencies between sub-fertile (case) and fertile (control) groups, odds ratios (ORs) and confidence intervals (95% CIs) were used. If the *P*-value was less than 0.05, it was considered statistically significant. SPSS Version 22 for Mac was used to conduct statistical analysis.

## Results

In the current study, we considered the fertile group as men who had at least one child and presented normal semen analysis parameters [volume: 1.5 ml, sperm count: 15 million spermatozoa/ml; normal forms: 4%; progressive motility: 32%; total motility (progressive + nonprogressive): 40%, according to WHO guideline 2010], and we considered the sub-fertile group as those who failed to have children after minimum 1 year of regular unprotected sexual intercourse and at least had one abnormal sperm parameter under WHO (2010) criteria. Thus, subjects were divided into two groups: a control group (fertile, n = 44) and a case group (sub-fertile, n = 67).

The age variance of the sub-fertile and fertile groups did not differ significantly in the study population (*P* = 0.225). The semen parameters, on the other hand, revealed significant differences between fertile and sub-fertile groups in the mean percentage of sperm concentration, total motility, and morphologically normal spermatozoa (*P* = 0.0001) (Table 2).

**Table 2:** Comparison of age and the semen analysis parameters between the control group (Fertile) and study group (Sub-fertile)

Parameter	Fertile (n = 44) Median	Sub-fertile (n = 67) Median	*P*-value
Age (years)	34 (26–48)	34 (26–48)	0.225
Sperm concentration (million/ml)	78.5 (17–185)	28 (0.6–135)	** < 0.0001**
Total Motility (%)	67.5 (44–90)	50 (2–88)	** < 0.0001**
normal morphology of spermatozoa (%)	24.5(20–30)	15 (0–28)	** < 0.0001**

Bold means significant association

### Genotypes and allele frequency

A total of twelve SNPs in the *MT-CO3* gene: rs2248727, rs7520428, rs3134801, rs9743, rs28358272, rs2853824, rs2856985, rs2854139, rs41347846, rs28380140, rs3902407, and 28,411,821, fourteen SNPs in the *MT-ATP6*: rs2001031, rs2000975, rs2298011, rs7520428, rs9645429, rs112660509, rs6650105, rs6594033, rs6594034, rs6594035, rs3020563, rs28358887, rs2096044, and rs9283154, and ten SNPs in the *MT-ATP8*: rs9285835, rs9285836, rs9283154, rs8179289, rs121434446, rs1116906, rs2153588, rs1116905, rs1116907, and rs3020563 were detected in the case and control groups at different nucleotide positions. (Tables 3, S1, 4, S2, 5, S3).

**Table 3:** Genotypes of CO3 polymorphisms between sub-fertile patients and controls

SNP	Contig position	Protein position	Amino acid change	Genotype	Sub-fertile	Fertile	*P*-value
rs2248727 T > C	9540	Leu112	Synonymous variant	TT	59	34	0.1315
				TC	0	0	
				CC	8	10	
rs7520428 A > G	634,390	–	–	AA	67	38	** < 0.0001**
				AG	0	0	
				GG	0	6	
rs3134801 T > C	9950	Val248	Synonymous variant	TT	67	43	0.1560
				TC	0	0	
				CC	0	1	
rs9743 T > C	9698	Leu164	Synonymous variant	TT	63	43	0.3221
				TC	0	0	
				CC	4	1	
rs28358272 C > T	9449	Tyr81	Synonymous variant	CC	65	44	0.1540
				CT	0	0	
				TT	2	0	
rs2853824 A > G	9347	Leu47	Synonymous variant	AA	67	43	0.1560
				AG	0	0	
				GG	0	1	
rs2856985 G > A	9755	Glu183	Synonymous variant	GG	67	43	0.1560
				GA	0	0	
				AA	0	1	
rs2854139 C > T	9818	His204	Synonymous variant	CC	67	44	0.1560
				CT	0	0	
				TT	0	0	
rs41347846 T > C	10,034			TT	67	44	0.1560
				TC	0	0	
				CC	0	0	
rs28380140 A > G	9377	Trp57	Synonymous variant	AA	67	43	0.1560
				AG	0	0	
				GG	0	1	
rs3902407 T > C				TT	67	44	0.1560
				TC	0	0	
				CC	0	0	
rs28411821 T > A,C	9824	Leu206	Synonymous variant	TT	67	43	0.1560
				TC	0	0	
				CC	0	1	

Bold means significant association

**Table 4:** Genotypes of ATP6 polymorphisms between sub-fertile patients and controls

SNP	Contig position	Protein position	Amino acid change	Genotype	Sub-fertile N (%)	Fertile N (%)	*P*-value
rs2001031 A > G	8860	Thr112Ala	Missense variant	AA	0	0	-
				AG	0	0	
				GG	67	44	
rs2000975 A > G	8701	Thr59Ala	Missense variant	AA	50	33	1.0000
				AG	3	1	
				GG	14	10	
rs2298011 A > G	9180	Val218	Synonymous variant	AA	67	43	0.3964
				AG	0	1	
				GG	0	0	
rs7520428 A > G	634,390	–		AA	67	38	** < 0.0001**
				AG	0	1	
				GG	0	5	
rs9645429 G > A, C	634,224	–		GG	63	43	0.3221
				GA	0	0	
				AA	4	1	
rs112660509 T > A,C	633,824	–		TT	64	42	0.7155
				TC	2	0	
				CC	1	2	
rs6650105 G > A,T	633,887	–		GG	64	43	1.0000
				GA	2	0	
				AA	1	1	
rs6594033 T > A,C	634,112	–		TT	66	43	0.5647
				TC	1	0	
				CC	0	1	
rs6594034 A > C,T	634,229	–		AA	65	43	0.6497
				AC	2	0	
				CC	0	1	
rs6594035 T > A,C	634,244	–		TT	65	42	0.6497
				TC	2	2	
				CC	0	0	
rs3020563 A > G	8566	Gln67	Synonymous variant	AA	67	43	0.1560
				AG	0	0	
				GG	0	1	
rs28358887 G > A,T	8994	Leu156	Synonymous variant	GG	66	43	1.0000
				GA	0	1	
				AA	1	0	
rs2096044 T > A,C,G	634,337	–		TT	66	44	1.0000
				TC	1	0	
				CC	0	0	
rs9283154 A > C,G,T	633,714	–		AA	66	44	1.0000
				AG	1	0	
				GG	0	0	

Bold means significant association

**Table 5:** Genotypes of ATP8 polymorphisms between sub-fertile patients and controls

SNP	Contig position	Protein position	Amino acid change	Genotype	Sub-fertile N (%)	Fertile N(%)	*P*-value
rs9285835 T > A, C	633,624	–		TT	62	41	1.0000
				TC	4	3	
				CC	1	0	
rs9285836 T > C	633,630	–		TT	62	40	0.7565
				TC	4	3	
				CC	1	1	
rs9283154 A > C,G,T	633,714	–		AA	62	41	1.0000
				AG	5	3	
				GG	0	0	
rs8179289 A > C,G,T	633,561	–		AA	60	40	1.0000
				AG	5	2	
				GG	2	2	
rs121434446 G > A	8392	Trp9	Synonymous variant	GG	67	44	
				GA	0	0	
				AA	0	0	
rs1116906 A > G	8460	Asn32Ser	Missense variant	AA	67	43	0.1560
				AG	0	0	
				GG	0	1	
rs2153588 C > T	633,672	–		CC	63	43	1.0000
				CT	4	0	
				TT	0	1	
rs1116905 C > A,T	8428	Phe21Leu	Missense varian	CC	67	43	0.1560
				CT	0	0	
				TT	0	1	
rs1116907 C > T	8468	Leu35	Synonymous variant	CC	67	43	0.1560
				CT	0	0	
				TT	0	1	
rs3020563 A > G	8566	Ile14Val	Synonymous variant	AA	67	43	0.1560
				AG	0	0	
				GG	0	1	

Nine of the SNPs detected in the *MT-CO3* gene, 4 of the SNPs in the *MT-ATP6* gene and 2 of the SNPs in the *MT-ATP8* gene were synonyms variants. (Tables 3, 4, 5), whereas the following SNPs of the *MT-ATP6* (rs2001031, rs2000975) and MT*-ATP8* (rs1116906, rs1116905) were located to cause a missense variation in the coding protein.

**Fig. 2:**
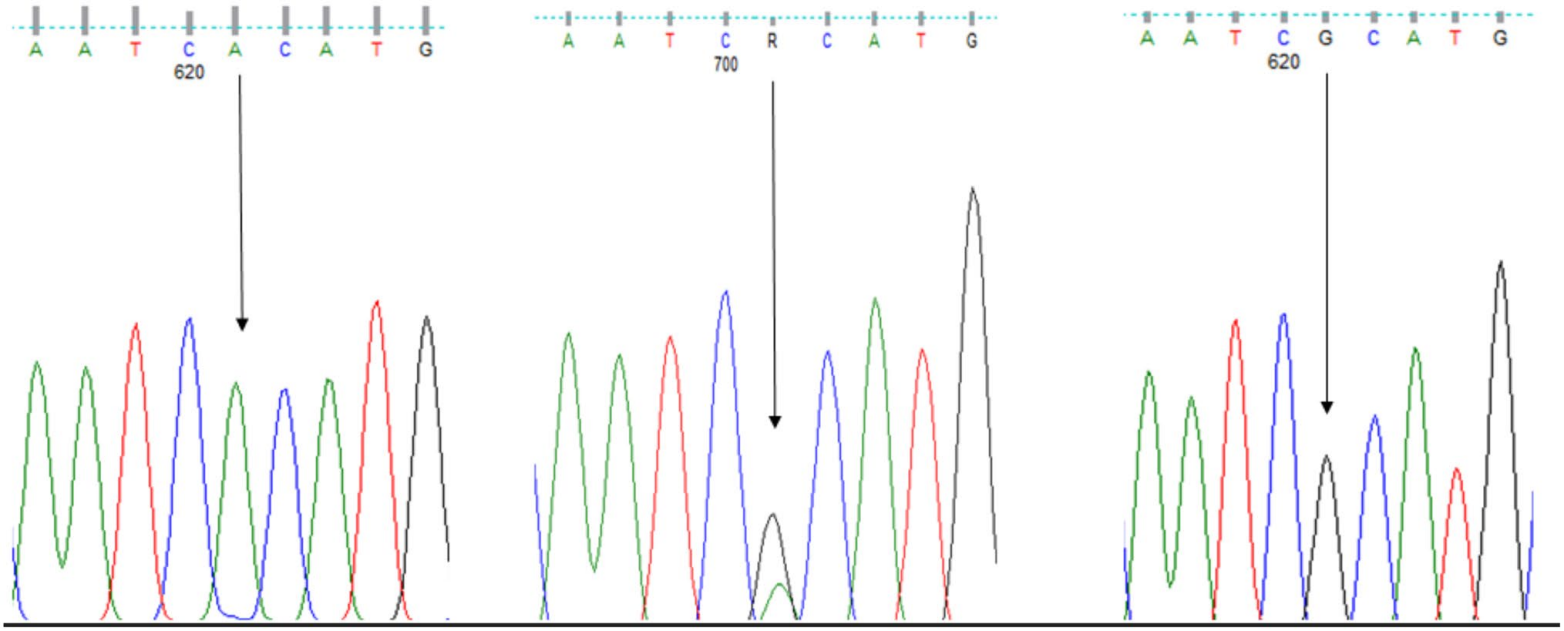
Sequencing electropherogram results (AA, AG, GG) of the rs7520428 of *MT-CO3.* The nucleotide transition at position 634,390 (A > G)

To determine whether the variations of *MT-CO3, MT-ATP6* and *MT-ATP8* were related to sub-fertility, we compared each of the genotypes and allele frequencies between the case and control groups. RS7520428 was found in both *MT-CO3* and *MT-ATP6* genes and both showed a significant difference in the genotype’s frequency test between sub-fertile and fertile groups (Figs. 1, 2). The rest of the SNPs showed no statistically significant association in frequencies of genotypes and alleles between the present MT-SNPs and male sub-fertility. Furthermore, the Hardy–Weinberg genotype frequency test was performed on all SNPs. Each of these SNPs deviated from HWE in a significant way (*P <* 0.0001). However, there was no statistically significant difference between sub-fertile males with asthenozoospermia, oligozoospermia, teratozoospermia, asthenoteratozoospermia, oligoasthenoteratozoospermia, and oligoteratozoospermia and those who were fertile (*P >* 0.05).

We evaluated the genotype and allele frequencies of *MT-CO3, MT-ATP6,* and *MT-ATP8* in sub-fertile and fertile groups to see if they were linked to male sub-fertility. Among the 12 *MT-CO3*, 14 *MT-ATP6*, and 10 *MT-ATP8* detected single nucleotide polymorphisms (SNPs), only the rs7520428 in the *MT-CO3* and *MT-ATP6* showed a statistically significant difference between sub-fertile and fertile groups in the genotypes and allele’s frequency test (P < 0.0001 for both).

## Discussion

The mitochondrion is the powerhouse of eukaryotic cells because of its capacity to create ATP through oxidative phosphorylation [21]. The mitochondrial function as an energy source is important for sperm function. It was reported that reduced sperm motility is linked to abnormalities in sperm mitochondrial ultrastructure [22].

Any abnormality in mitochondrial respiratory activity will adversely affect the process of spermatogenesis, particularly the advancement of pachytene phases during meiosis and sperm production. Spermatogenic cells will undergo mitochondrial respiratory failure if large amounts of pathogenic mutant mtDNA accumulate in the testicular tissue. Any reduction in the mitochondrion's ability to generate energy during spermatogenesis causes meiotic arrest. Spermatocytes with a lack of oxygen will most likely not complete meiosis and will be killed by apoptosis [23]. Spermatozoa with defective mitochondria produce insufficient ATP and have elevated levels of reactive oxygen species (ROS) or free radicals. In an imbalanced system, the production of ROS and free radicals would cause significant damage to the mitochondria and mtDNA, affecting sperm motility and finally leading to male infertility [24]. More specifically, an inadequate molecular profile of all types of male infertility still remains. Since mitochondrial biogenesis is essential for the proper motility of the sperm, any variations in mtDNA, whether quantitative or qualitative, influence the spermatozoa's cellular functioning. Specific mtDNA deletions in sperm have been linked to poor sperm function. Multiple 7345 and 7599 bp mtDNA deletions have been linked to poor sperm motility [25]. Moreover, studying genetic variations such as SNPs might be a helpful genetic analysis to understand the molecular bases of idiopathic infertility in males. In many studies, certain SNPs have shown an association with certain disorders such as cancer and infertility [24].

This work aimed to investigate whether there was an association between polymorphisms in the mitochondrial genes MT-CO3, MT-ATP6 and MT-ATP8 and male sub-fertility. Accordingly, we performed direct sequencing to check for polymorphisms in the MT-CO3, MT-ATP6, and MT-ATP8 genes in sub-fertile and fertile males, 12 SNPs have been identified in the MT-CO3 gene (rs2248727, rs7520428, rs3134801, rs9743, rs28358272, rs2853824, rs2856985, rs2854139, rs41347846, rs28380140, rs3902407, and 28,411,821), 14 SNPs in the MT-ATP6 (rs2001031, rs2000975, rs2298011, rs7520428, rs9645429, rs112660509, rs6650105, rs6594033, rs6594034, rs6594035, rs3020563, rs28358887, rs2096044, and rs9283154) and 10 SNPs in the MT-ATP8 (rs9285835, rs9285836, rs9283154, rs8179289, rs121434446, rs1116906, rs2153588, rs1116905, rs1116907, and rs3020563).

Amongst reported SNPs of the previously mentioned mitochondrial genes, rs7520428 was found in both MT-CO3 and MT-ATP6 genes and both showed a significant difference in the genotype’s frequency test between sub-fertile and fertile groups. In addition, the allele frequency of rs7520428 SNP (A634390G) in MT-CO3 and MT-ATP6 showed a significant association with male subfertility (*P <* 0.0001). Furthermore, the OR of the rs7520428 SNP was linked to a 40-fold greater risk of subfertility in men compared to fertile ones. This could indicate that expanding the number of wild-type alleles (A) or reducing the number of mutant alleles (G) at A634390G can effectively protect male fertility, whereas increasing the number of G alleles or decreasing A alleles can develop male subfertility. This genetic alteration could be related to male sub-fertility which required further investigation to understand its role in the function of the protein outcome.

The current findings showed that nine of the SNPs detected in the MT-CO3 gene, 4 of the SNPs in the MT-ATP6 gene and 2 of the SNPs found in the MT-ATP8 gene were synonyms variants. Synonymous mutations have been hypothesized to have an impact on gene control and the establishment of disorders [26]. It has been found that synonymous variants possibly affect mRNA stability [26]. As a result, functional investigations on these synonymous variants in mtDNA are needed to reveal their potential involvement in sperm function and male sub-fertility.

The rs2001031 (A8860G) and rs2000975 (A8701) are missense mutations that alter threonine to alanine and they were significantly associated with subfertility. On the other hand, the following SNPs were detected in the current study and were reported to be pseudogenes: MT-CO3 (rs7520428) MT-ATP6 (rs7520428, rs9645429, rs112660509, rs6650105, rs6594033, rs6594034, rs6594035, rs2096044, rs9283154) MT-ATP8 (rs9285835, rs9285836, rs9283154, rs8179289, rs2153588).

Mughal and his group have reported that there was a significant association between the 15 bp deletion (at position 9390 to 9413) of cytochrome C oxidase III and human male infertility (*P* = 0.033) [27]. In our study, the RS2000975 (A8701C, G) showed no significant association with male sub-fertility in the Genotype frequency (*P* = 1.0000), on the other hand, a previous study has reported a significant association between A8701G variants and increased risk of fertilization failure [28]. The inconsistent findings are attributed to the genetic variations as well as the subtype of infertility in the study populations. For instance, a recent study reported a significant correlation between polymorphisms of the MT-CYB gene and sub-fertility in men. Particularly, rs527236194, rs28357373 and rs41504845 variants were found significantly related to the sub-fertility group [29].

In conclusion, further research on a larger sample size of different populations is needed to emphasize the role of the reported SNPs in male sub-fertility. In addition, functional studies will be very helpful in understanding the molecular impact of each specific SNP on the function of the protein outcome and the mitochondrial efficiency that may explain their role in sperm function.

## Supplementary Information

Below is the link to the electronic supplementary material.

Supplementary file1 (DOCX 28 kb)
