# Supplementary material for: A lack of a definite correlation between male sub-fertility and single nucleotide polymorphisms in sperm mitochondrial genes MT-CO3, MT-ATP6 and MT-ATP8
Source: Mol Biol Rep. 2022 Sep 6;49(11):10229–38. doi: 10.1007/s11033-022-07884-2 (PMC9618475; doi:10.1007/s11033-022-07884-2)
Supplement: Supplementary file 1 — Supplementary file1 (DOCX 28 kb) [file 11033_2022_7884_MOESM1_ESM.docx]

**Supplementary**

**Table S1 Alleles frequency of CO3 polymorphisms between sub-fertile patients and controls.**

| **SNP** | **Contig position** | **Protein position** | **Alleles** | **Sub-fertile, %** | **Fertile, %** | **OR (95% CI)*** | ***P*-value** |
| --- | --- | --- | --- | --- | --- | --- | --- |
| **rs2248727 T>C** | 9540 | Leu112 | T | 118  (53%) | 68 (31%) | 2.169  (1.054 to 4.466) | 0.0516 |
|  |  |  | C | 16 (7%) | 20 (9%) |  |  |
| **rs7520428 A>G** | 634390 | - | A | 134 (60%) | 76 (34%) | 43.954  (2.565 to 753.29) | **< 0.0001** |
|  |  |  | G | 0 (0%) | 12 (6%) |  |  |
| **rs3134801 T>C** | 9950 | Val248 | T | 134 (60%) | 86 (39%) | 7.775  (0.3685 to 164.02) | 0.3044 |
|  |  |  | C | 0 (0%) | 2 (1%) |  |  |
| **rs9743**  **T>C** | 9698 | Leu164 | T | 126 (57%) | 86 (39%) | 0.3663  (0.07590 to 1.768) | 0.3328 |
|  |  |  | C | 8 (3%) | 2 (1%) |  |  |
| **rs28358272 C>T** | 9449 | Tyr81 | C | 130 (58%) | 88 (40%) | 0.1638  (0.008706 to 3.083) | 0.2628 |
|  |  |  | T | 4 (2%) | 0 (0%) |  |  |
| **rs2853824 A>G** | 9347 | Leu47 | A | 134 (60%) | 86 (39%) | 7.775  (0.3685 to 164.02) | 0.3044 |
|  |  |  | G | 0 (0%) | 2 (1%) |  |  |
| **rs2856985 G>A** | 9755 | Glu183 | G | 134 (60%) | 86 (39%) | 7.775  (0.3685 to 164.02) | 0.3044 |
|  |  |  | A | 0 (0%) | 2 (1%) |  |  |
| **rs2854139 C>T** | 9818 | His204 | C | 134 (60%) | 88 (40%) | - | - |
|  |  |  | T | 0 (0%) | 0 (0%) |  |  |
| **rs28380140 A>G** | 9377 | Trp57 | A | 134 (60%) | 86 (39%) | 7.775  (0.3685 to 164.02) | 0.3044 |
|  |  |  | G | 0 (0%) | 2 (1%) |  |  |
| **rs3902407 T>C** |  |  | T | 134 (60%) | 88 (40%) | - | - |
|  |  |  | C | 0 (0%) | 0 (0%) |  |  |
| **rs28411821 T>A,C** | 9824 | Leu206 | T | 134 (60%) | 86 (39%) | 7.775  (0.3685 to 164.02) | 0.3044 |
|  |  |  | C | 0 (0%) | 2 (1%) |  |  |
| **rs41347846 T>C** |  |  | T | 134 (60%) | 88 (40%) | - | - |
|  |  |  | C | 0 (0%) | 0 (0%) |  |  |

**Table S2 Alleles frequency of ATP6 polymorphisms between sub-fertile patients and controls.**

| **SNP** | **Contig position** | **Protein position** | **Alleles** | **Sub-fertile, %** | **Fertile, %** | **OR (95% CI)*** | **P value** |
| --- | --- | --- | --- | --- | --- | --- | --- |
| **rs2001031**  **A>G** | 8860 | Thr112Pro | A | 0 (0%) | 0 (0%) | - | - |
|  |  |  | G | 134 (60%) | 88 (40%) |  |  |
| **rs2000975**  **A>G** | 8701 | Thr59Pro | A | 103 (46%) | 67 (30%) | 1.041  (0.5526 to 1.963) | 0.9001 |
|  |  |  | G | 31 (14%) | 21 (10%) |  |  |
| **rs2298011**  **A>G** | 9180 | Val218 | A | 134 (60%) | 87 (39%) | 4.611  (0.1856 to 114.58) | 0.8319 |
|  |  |  | G | 0 (0%) | 1 (1%) |  |  |
| **rs7520428**  **A>G** | 634390 | - | A | 134 (60%) | 77 (35%) | 39.916  (2.318 to 687.23) | **0.0001** |
|  |  |  | G | 0 (0%) | 11(5%) |  |  |
| **rs9645429**  **G>A, C** | 634224 | - | G | 126 (57%) | 86 (38%) | 0.3663  (0.07590 to 1.768) | 0.3328 |
|  |  |  | A | 8 (4%) | 2 (1%) |  |  |
| **rs112660509 T>A,C** | 633824 | - | T | 130 (59%) | 84 (37%) | 1.548  (0.3767 to 6.359) | 0.8087 |
|  |  |  | C | 4 (2%) | 4 (2%) |  |  |
| **rs6650105**  **G>A, T** | 633887 | - | G | 130 (59%) | 86 (38%) | 0.7558  (0.1354 to 4.219) | 0.7489 |
|  |  |  | A | 4 (2%) | 2 (1%) |  |  |
| **rs6594033**  **T>A, C** | 634112 | - | T | 132 (59%) | 86 (39%) | 3.070  (0.2740 to 34.397) | 0.7168 |
|  |  |  | C | 1 (1%) | 2 (1%) |  |  |
| **rs6594034**  **A>C, T** | 634229 | - | A | 132 (59%) | 86 (39%) | 1.535  (0.2121 to 11.108) | 0.6690 |
|  |  |  | C | 2 (1%) | 2 (1%) |  |  |
| **rs6594035**  **T>A, C** | 634244 | - | T | 132 (59%) | 86 (39%) | 1.535  (0.2121 to 11.108) | 0.6690 |
|  |  |  | C | 2 (1%) | 2 (1%) |  |  |
| **rs3020563**  **A>G** | 8566 | Gln67 | A | 134 (60%) | 86 (39%) | 7.775  (0.3685 to 164.02) | 0.3044 |
|  |  |  | G | 0 (0%) | 2 (1%) |  |  |
| **rs28358887 G>A,T** | 8994 | Leu156 | G | 132 (59%) | 87 (39%) | 0.7586  (0.06771 to 8.500) | 0.8221 |
|  |  |  | A | 2 (1%) | 1 (1%) |  |  |
| **rs2096044 T>A,C,G** | 634337 | - | T | 133 (59%) | 88 (40%) | 0.5028  (0.02024 to 12.493) | 0.4167 |
|  |  |  | C | 1 (1%) | 0 (0%) |  |  |
| **rs9283154 A>C,G,T** | 633714 | - | A | 133 (59%) | 88 (40%) | 0.5028  (0.02024 to 12.493) | 0.4167 |
|  |  |  | G | 1 (1%) | 0 (0%) |  |  |

**Table S3 Alleles frequency of ATP8 polymorphisms between sub-fertile patients and controls.**

| **SNP** | **Contig position** | **Protein position** | **Alleles** | **Sub-fertile, %** | **Fertile, %** | **OR (95% CI)*** | ***P*-value** |
| --- | --- | --- | --- | --- | --- | --- | --- |
| **rs9285835**  **T>A, C** | 633624 | - | T | 128 (58%) | 85 (38%) | 0.7529  (0.1833 to 3.094) | 0.9625 |
|  |  |  | C | 6 (3%) | 3 (1%) |  |  |
| **rs9285836**  **T>C** | 633630 | - | T | 128 (58%) | 83 (37%) | 1.285  (0.3799 to 4.348) | 0.9296 |
|  |  |  | C | 6 (3%) | 5 (2%) |  |  |
| **rs9283154 A>C,G,T** | 633714 | - | A | 129 (58%) | 85 (38%) | 0.9106  (0.2120 to 3.912) | 0.8997 |
|  |  |  | G | 5 (3%) | 3 (1%) |  |  |
| **rs8179289 A>C,G,T** | 633561 | - | A | 125 (56%) | 82 (37%) | 1.016  (0.3485 to 2.963) | 0.9764 |
|  |  |  | G | 9 (4%) | 6 (3%) |  |  |
| **rs121434446 G>A** | 8392 | Trp9 | G | 134 (60%) | 88 (40%) | - |  |
|  |  |  | A | 0 (0%) | 0 (0%) |  |  |
| **rs1116906**  **A>G** | 8460 | Asn32Ser | A | 134 (60%) | 86 (39%) | 7.775  (0.3685 to 164.02) | 0.3044 |
|  |  |  | G | 0 (0%) | 2 (1%) |  |  |
| **rs2153588**  **C>T** | 633672 | - | C | 130 (58%) | 86 (39%) | 0.7558  (0.1354 to 4.219) | 0.7489 |
|  |  |  | T | 4 (2%) | 2 (1%) |  |  |
| **RS. 1116905 C>A,T** | 8428 | Phe21Leu | C | 134 (60%) | 86 (39%) | 7.775  (0.3685 to 164.02) | 0.3044 |
|  |  |  | T | 0 (0%) | 2 (1%) |  |  |
| **rs1116907**  **C>T** | 8468 | Leu35 | C | 134 (60%) | 86 (39%) | 7.775  (0.3685 to 164.02) | 0.3044 |
|  |  |  | T | 0 (0%) | 2 (1%) |  |  |
| **rs3020563**  **A>G** | 8566 | Ile14Val | A | 134 (60%) | 86 (39%) | 7.775  (0.3685 to 164.02) | 0.3044 |
|  |  |  | G | 0 (0%) | 2 (1%) |  |  |
